# Computer- and Robot-Assisted Therapies to Aid Social and Intellectual Functioning of Children with Autism Spectrum Disorder

**DOI:** 10.3390/medicina55080440

**Published:** 2019-08-05

**Authors:** Joan DiPietro, Arpad Kelemen, Yulan Liang, Cecilia Sik-Lanyi

**Affiliations:** 1 Department of Organizational Systems and Adult Health, School of Nursing, University of Baltimore, MD 21201, USA; 2 Department of Family and Community Health, School of Nursing, University of Baltimore, MD 21201, USA; 3 Department of Electrical Engineering and Information Systems, University of Pannonia, 8200 Veszprem, Hungary

**Keywords:** autism spectrum disorders, intervention psychosocial/behavioral, school-age children, robots, computers, therapy

## Abstract

*Background and objectives*: Children with autism spectrum disorder (ASD) experience challenges with social interactions, a core feature of the disorder. Social skills therapy has been shown to be helpful. Over the past several years, computer-assisted and robot-assisted therapies have been infiltrating the social skills teaching environment. Rapid progress in the field of technology, especially in the robotics area, offers tremendous possibilities for innovation and treatment or even education for individuals with ASD. This paper’s purpose is to drive awareness of these innovative interventions in order to support the social lives of children with ASD. The aims of the paper are identifying (1) the types of Information Technology platforms that are being evaluated in computer and robot-assisted therapies for children with ASD; (2) the various disciplines or professions studying and utilizing these computer and robot-assisted social skill therapies; (3) the outcomes being evaluated in each trial; and (4) if results demonstrate benefits to children with autism. *Materials and Methods*: PubMed, CINAHL, Science Direct, and Web of Science databases were searched for clinical trials published over the past five years. Search terms incorporated the subject intersection of autism, and computer or robot-assisted therapy. Results were mined for pediatric populations only and study designs establishing controlled comparisons. *Results*: Eighteen unique international studies were identified that utilize robot interventions (11 studies) and serious computer game interventions (seven studies). Most demonstrated promising results in improving outcomes for children with ASD. Study implications reveal a rapidly evolving assistive technology for ASD social skills therapy. *Conclusions*: These interventions show considerable promise, but more effectiveness and cost effectiveness research of high quality should be carried out with larger numbers of children. Also, further studies are necessary to evaluate these technologies’ effectiveness amongst adults with ASD and within unique subsets of the higher functioning autism population.

## 1. Introduction

Social skill deficits are a core challenge for those diagnosed with autism spectrum disorders (ASD) [1]. Therapies that address these challenges are available; however, a single program or regimen is not effective for all individuals with ASD. This may be due to large variations in skill level, cognitive ability, coping ability, and the type and number of specific challenges manifested in each individual with this spectrum disorder [1,2]. The employment of educational robots for children with ASD, in particular, is rapidly increasing, and evidence is growing documenting its positive impact in addressing core features of ASD, including communication and social relationships. It is of theoretical and practical value for educators and researchers to have an overview of the literature describing in which conditions educational robotics or other interactive computerized therapies (ICT) may be most effective when applied to ASD.

Approximately four in 10 people with autism have a learning disability, which is a lifelong disorder diagnosed in childhood [3]. Although ASD is not a learning disability, it does affect learning. This is why it is necessary to study ASD and learning disability together. Learning disabilities may affect activities such as acquisition, organization, retention, and understanding or use of verbal or nonverbal information [4,5,6,7]. These distinguish from global intellectual deficiency, which describes those who otherwise demonstrate at least average abilities essential for thinking and/or reasoning. Learning disabilities can cause problems with speaking, reading, writing, math, concentration, organization, time, social interactions, or speech comprehension. Often, children have more than one kind of learning disability, such as attention deficit hyperactivity disorder (ADHD), which can make learning even more of a challenge [7].

Despite the abundance of literature in the area of ASD, there is limited research on ASD about users with learning disability. Some guidelines suggest that provision of therapies in early childhood offer the child with ASD enhanced benefits over alternative therapies offered later in life [8]. Identifying ASD as early as possible and providing evidence-based therapies known to produce positive outcomes is therefore crucial. One of the most important therapies to offer the child with ASD addresses social skill competency, pivotal to help those with ASD overcome social/communication challenges, a core deficit in this disorder [1,2,8,9,10,11,12,13]. These programs are not standardized in their curriculum and are offered by various professional and nonprofessional instructors and therapists [14,15,16,17]. Programs differ by skills taught, participant age-bands, medium utilized (e.g., video modeling, in-person sessions, and web-based interactive sessions), duration of therapy, and length of sessions. Social skill therapies are offered as individual or group sessions and are available in schools, clinician offices, public meeting places, or live electronic video environments.

Social skills therapies include interventions such as play therapy, didactic social skills instructions, cognitive behavioral therapy, modeling, and practice therapies [13,14,15]. Behavioral interventions that address atypical or disruptive actions such as stereotypies, anxiety tantrums, aggressive behaviors, and defiance may also be considered social skill training, as the ability to self-manage one’s negative behaviors assists in social acceptance. Speech therapies, offering receptive and expressive instruction, including pragmatic skills, may be considered a form of social skills training as well, as it teaches effective communication with others. Multiple professional disciplines provide differing therapies to assist the child with ASD. A team approach is commonly required to concurrently address multiple issues [15,16]. Comprehensive treatment by a team of professionals including teachers, speech therapists, occupational and physical therapists, mental health providers, behaviorists, social workers, and medical clinicians, along with parents, other family members, and caregivers, all participate to influence the current and future potential of these children.

Rapid progress in the field of technology, especially robotics, offers tremendous possibilities for innovation in treatment or even education for individuals with ASD. Recent studies have shown that computer-based learning has made a huge contribution for children with intellectual disability. It enables pupils to take charge of their own learning, as they find stimulation through “enjoyable repetition” and a gradual increase in level of challenge [18]. Blamires argues that enabling technology provides access to educational opportunities, life experiences, and facilitates engagement with knowledge and people [19]. The assistive technologies combine speech, pictures, words, and animation in interactive ways to structure concepts that suit the level of understanding of learners and their interest [20]. More importantly, computer-based instruction and game-based learning can make a very real contribution to teaching essential life skills for those with ASD [21].

Computer-assisted and robot-assisted therapy is infiltrating the social skills teaching environment, being trialed or incorporated into therapy by a variety of professions to help teach the child with ASD [16,17,22]. Validation of effectiveness of computer-aided therapies to teach social skills is warranted to justify the quality of these interventions. Useful technologies will likely proliferate further into therapy regimens, offering new models and assistance to those who serve these children and their families. The key question is where can we find the appropriate tools and assistive technologies, e.g., computer-assisted therapies to support those with ASD and learning disabilities? Furthermore, how do we prepare the learning information such that teachers, therapists, and parents may easily find and use them for children with multiple disabilities?

In this paper, we identify the types of information technology platforms and evaluate the computer- and robot-assisted therapies in regards to their appropriateness for children with ASD and learning disabilities. The purpose of this literature review includes the following aims. (1) To answer the research question: What types of Information Technology (IT) platforms are being evaluated in computer and robot-assisted therapies for children with ASD? (2) To identify the various disciplines studying computer and robot-assisted social skill therapies. (3) To identify the outcomes being evaluated in each trial. (4) To determine if results demonstrate benefits to children with autism.

We systematically collected information on available review literature on the topics of ASD, social skills, communication training/interventions, and technology [23,24,25,26,27]. Based on these reviews, we identified a gap in the existing literature/research on the topic of what computer game and robot-assisted therapies are being used to aid social and intellectual functioning of children with ASD.

## 2. Methods

A thorough literature search in PubMed, CINAHL, ScienceDirect, and Web of Science (WoS) databases was conducted to find controlled clinical trials (measuring comparison to a control arm) on the subject intersection of autism, computer-assisted therapy, and children. We were interested in the types of computerized tools being utilized for social skill development in children with ASD—namely, what information technology platforms were being evaluated in controlled studies and reported in peer-reviewed literature? We set a search period for studies published between January 2015 and January 2019. Search words included MeSH terms “autism*” or “development* delay” and “computer-assisted” or “computer-aided” or “robotics” or “game” or “gaming”. A list of 619 records was identified. Duplicates were then removed, reducing the search results to 374 specific records. Two-hundred-and-nine records were then eliminated due to limiters for English language, research intervention, and open or free downloadable access.

**Figure 1:**
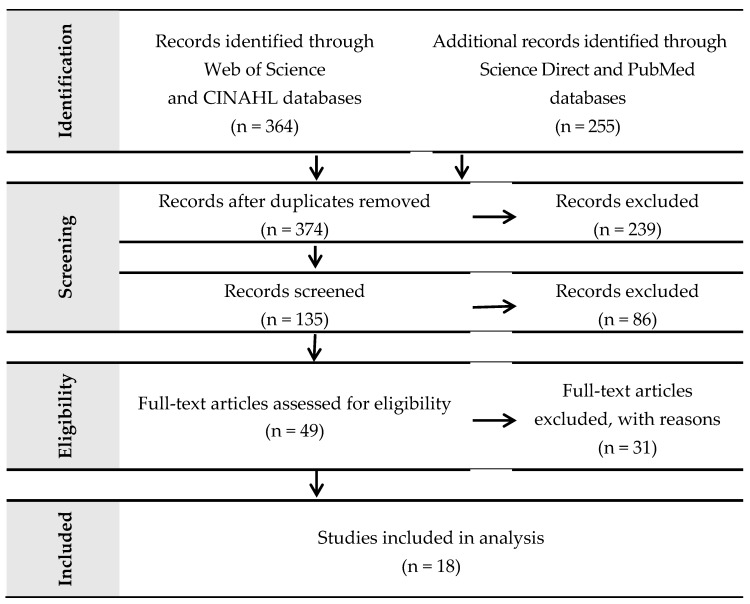
Choosing the analyzed papers based on PRISMA flowchart.

Abstracts of the resulting 135 records were then screened for relevancy to our research goals. 86 records were deemed inappropriate due to non-autistic study populations. Next, two different researchers conducted full text article reviews of the remaining 49 records. Thirty-one records were eliminated due to the following exclusion reasons; not a child study, not evaluating a social skills outcome, or not exercising a controlled study design (no comparison to control arm). The resulting 18 records meeting all eligibility criteria are evaluated in this literature review. Figure 1 shows the flowchart of choosing methodology based on PRISMA flowchart [28]. PRISMA is an evidence-based minimum set of items for reporting in systematic reviews and meta-analyses.

We then classified each study into two categories based on “Type of IT intervention Platform” and the social skill “Therapy Target”. This effort was intended to organize the information and evaluate it as a whole, and to recognize commonality & uniqueness across variables at a glance (see Table A1 for the summarized computer-assisted therapies for children with ASD). IT platform types were noted to be either robot-delivered interventions or serious computer game interventions. If a study met both intervention domains, it was grouped by the component being investigated. For example, in a study involving a robot participating in a computer game with the child with ASD evaluating the child’s reaction to the robot, this was assigned to Robots.

## 3. Results

IT Platform categorization activity revealed two mediums being utilized, with eleven studies being robot interventions and seven being serious computer game interventions. Summarizing each of the reviewed studies within these two domains helps reveal the penetration of these computerized assistance platforms and their effectiveness as a group in teaching children with ASD social skills.

### 3.1. Robot Interventions

The increasing deployment of robots in recent decades has inspired new boundaries for different therapies. Animal-like robots have received especially notable acceptance in therapeutic settings. Robots were featured as the intervention in eleven unique studies. A robot is a mechanical or virtual agent capable of moving independently and performing complex actions [29,30]. This paper mainly deals with humanoid robots. A humanoid robot is a robot with its overall appearance based on that of the human body [31].

To simulate an autonomous behavior model, researchers in New Zealand [19] used a semiautonomous parrot-inspired robot (KiliRo). Robot-supported therapy using adapted model–rival method was experimentally tested with nine children with ASD for five consecutive days in a clinical setting. Facial expressions of the children were analyzed when they contacted KiliRo using an application program interface called the Oxford emotion API (Application Programming Interface). The results showed signs that children with ASD were attracted and were happy to interact with the parrot-inspired robot.

The notion that children with ASD prefer robots as tutors to improve their social interaction and communication abilities is supported by recent studies. Indeed, the research focused on developing a very promising form of intervention called robot-assisted therapy. This therapy has a number of challenges, e.g., the necessary flexibility and adaptability to real unrestricted therapeutic settings. Pennisi et al. reviewed studies in the period of 2006 to 2016, asking if social robots could be a useful tool in autism therapy [23].

The most frequent deficiency to children with autism and mental disability is social attention, which includes the difficulty of focusing good visual attention. Di Nuovo and his colleagues [32] examined the use of a new deep learning neural network architectures to automatically determine whether a child focused on visual attention during a therapeutic session, indicating their commitment. They used the Nao humanoid robot for their research and have proposed the use of computer intelligence techniques to increase robot capabilities for greater adaptability and flexibility, enabling the robot to be integrated into any therapeutic environment, according to the specific needs of the therapist and the individual child. Their article represented a step forward in this direction, as the authors dealt with the problem of evaluating the child’s visual focal point from the low-resolution video footage of the robot camera.

A study by Huskens et al. utilized a robot-mediated intervention based on LEGO^®^ Therapy to study impact on collaborative play behavior [33,34]. The study took place in the Netherlands. The population included three sibling pairs, one sibling with ASD, the other without. The siblings had to be within 5 years in age from their brother/sister partner. The siblings with ASD all had IQs greater than 80. Sibling pairs were randomly assigned to different baseline lengths of three, four, or five sessions. The dependent/outcome variables included collaborative behaviors exhibited as (1) interaction initiations, (2) responses to questions or instructions from typically developing siblings, and (3) play together actions to achieve a common goal. During five 30-minute sessions once a week, a robot would instruct one child of the sibling pair to be the guide, the other the LEGO^®^ builder. The guide received the LEGO^®^ instruction booklet and the builder collected the LEGO^®^ bricks and put them together as instructed by the guide. The robot reinforced collaboration and offered prompts. The humanoid 57cm “social robot” in this trial was named NAO (Aldebaran Robotics, n.d.) [35]. Robot features included preprogrammed speech, a female voice, was Dutch-speaking, and able to move its arms, legs, and fingers. The robot had eyes and a mouth, but no nose. It was controlled by a human trainer using a laptop. Results demonstrated no statistically significant changes in collaborative behaviors in any of the three measurement targets for the children with ASD. Of interest is the social validity measure result, indicating that the children with ASD reported the robot sessions were less enjoyable than the nonrobot sessions while their typically developing siblings reported the opposite.

Social attention skills were studied with a robot intervention by Srinivasan et al. [36]. Thirty-six children with ASD aged 5–12 years participated in this randomized controlled pilot study evaluating the effects of novel movement-based interventions to current standards of care. In addition, human trainer vs. robot trainer effects were compared. Thirty-two intervention sessions were delivered over 8 weeks. The human trainers consisted of physical therapists, a kinesthesia graduate student, and 2 parents. The robot trainers were the humanoid robot NAO controlled mainly by a human trainer using a laptop and Rovio™ (WowWee) [37], a wi-fi enabled mobile webcam. All groups performed joint action based gross and/or fine motor activities that prompted social skills. The rhythm and robotic intervention groups incorporated movement-based games promoting both gross and fine motor skills. The comparison group utilized sedentary activities promoting fine motor skills. The rhythm and comparison groups demonstrated joint attention improvements. Social attention was improved most in the rhythm group, followed in sequence by the robot group and comparison group. The authors reported that rhythmic and whole-body interpersonal synchrony games led to high levels of social attention compared to sedentary activities. The robot intervention was noted to be hindered by technical limitations, suggesting advances in autonomy and contingent responding would make the robot a more effective tool to assist children with ASD.

In another similar study by Srinivasan et al. [38] utilizing the previously reported rhythm and robotic therapy intervention with NAO and Rovio, the outcome targets were repetitive behaviors and affective states in children with ASD. Repetitive, disruptive behaviors negatively impact social acceptance and thus are often addressed as part of social skills therapy. Following thirty-two 45-minute intervention sessions, rhythm and robotic therapy was compared to the standard of care intervention in 36 children with ASD, age 5–12 years. The outcomes targeted were frequencies of sensory, negative, and stereotyped behaviors. In addition, the duration of time the children displayed positive, negative, and interested effect was measured. Results indicated that early in the sessions, the rhythm and robot groups exhibited greater negative behaviors than the control group. The control group exhibited greater sensory behaviors. After training, the rhythm group reduced negative behaviors. The other groups did not. Affective state results indicated the rhythm and robotic groups demonstrated greater interested affect across all sessions. Negative affect was decreased and interested affect increased in the rhythm group after training. The robot intervention however displayed reduction in positive affect. The authors concluded their results suggest rhythm-based interventions are socially engaging treatment tools to address core challenges in children with ASD.

Taheri et al. [39] introduced clinical interventions with social humanoid robots in the treatment of autistic Iranian twins in a pilot study based on a single subject design experiment. The robot-assisted interventions for a pair of fraternal twins—one with HFA and one with LFA—are described. Either the robot and/or the therapist gave the instructions for each game to the children and their parents. The treatments were held in a 5 × 5 × 3 m room. The experimental setup was made up of one or occasionally two humanoid robots. In addition, there were two laptops, two cameras (for filming sessions), Microsoft Kinect Sensor, a video projector, and a whiteboard and chairs for all involved. Results of the 2.5-month robotic treatment demonstrated improvement in the HFA subject’s social and communication skills, while the LFA subject showed improvement in decreased stereotyped behaviors. While noting several study limitations, the researchers observed that robot group games had the potential to improve not only communication but social skills too.

In another research Taheri et al. [40] observed significantly increased verbal communications of paired-groups following a robot-assisted group games program. The six male participants with autism consisted of three pairs: (1) a pair of 7-year-old fraternal twins, one of whom with high-functioning and the other one with low-functioning autism; (2) two siblings with high-functioning ASD, one age 15 years, the other age 10 years; and (3) two high-functioning classmates aged 6 and 7 years old. The participants took part in games at each session in different modes: Robot–Child or Robot–Child–Peer/Parent/Therapist interactions. (i) Real-time imitation by the robot in upper body movements of the child (in Robot–Child mode). (ii) Teaching imitation/motor skills by the robot to the children through individual/paired-group exercise and dances (in Robot–Child and Robot–Child–Peer/Parent modes). (iii) Playing a real xylophone (in Robot–Child mode) pointing to far/near points and showing the cards/objects by the robot/child (in Robot–Child mode). (iv) Kinect-based recognition game and classification of animals, fruits, places, and objects by pointing to different baskets on the screen (in Robot–Child and Robot–Child–Parent modes). (v) Playing a developed Kinect-based virtual xylophone on the screen (in Child–Parent/Therapist modes).

Pour et al. [41] studied facial expression recognition in a 2-part study using a humanoid robot named “Mina”. The first part of the study measured reaction and acceptance of the robot’s facial responses by children with ASD. Fourteen Iranian children ages 3–7 years participated with a robot-acceptance rate of 78%. A second stage of the study compared the children’s performance reciprocating facial gestures modeled by the Mina robot versus by a human mediator. Results showed the subjects with ASD had better performance mimicking the human mediators than the robot.

A study by van Straten et al. [42] evaluated task performance and affective state outcomes of children with ASD aged 4–8 years playing puzzle games with a robot. The researchers studied the effects of the robot’s intonation and bodily appearance, noting that both impacted the children’s affective states but not their task performance. Researchers stated the robot’s human-like body appearance as compared to mechanical bodily appearance led to a higher degree of interest by the child in the child–robot interaction and that congruence of bodily appearance and intonation triggered a higher degree of happiness in the children.

David et al. [43] investigated use of a social robot in Cluj-Napoca, Romania. Five children with ASD participated in their research study. The researchers hypothesized that the more social cues the robot uses in child–robot interaction sessions (i.e., head orientation, pointing, and verbal indication), the better the child would perform maintaining joint attention. The results met the hypothesis.

A study by Desideri et al. [44] reported the results of a pilot test conducted using a social robot intervention targeting developmental and social skills. This study evaluated the educational sessions’ impact on engagement and learning achievement in two 9-year-old male children with ASD and intellectual disability. Results suggested that interaction with a social robot enhanced engagement and goal achievement in one participant while the 2nd participant demonstrated only enhanced goal achievement.

A creative study combining virtual reality technology and social robotics for tutoring children with ASD was undertaken by Saadatzi et al. [45]. Research subjects included three children with ASD, ages 6–8 years. The tutoring system featured a virtual teacher instructing sight words, and included a humanoid robot emulating a peer. Results indicated that participants acquired, maintained, and generalized 100% of the words explicitly instructed to them, made fewer errors while learning the words common between them and the robot peer, and vicariously learned 94% of the words solely instructed to the robot. Researchers observed that participants responded positively to the robot peer’s performance (e.g., “thank you” and “nice job”). One of the participants consistently greeted the robot and hugged it when the session was completed. Another participant began imitating the robot’s happy gestures. The researchers suggested that similar package may serve as a context under which learners can safely practice the performance of critical social responses.

So et al. [46] also utilized robot intervention in their research. They examined whether Chinese-speaking preschool children with ASD in their early childhood could catch up to the level of gestural production found in typically developing, age-matched children and whether they showed an increase in verbal imitation after the completion of robot-based training intervention. Comparison was made to a waitlist control group. Results were favorable during the trial and were maintained in delayed post-tests. The researchers concluded that robot-based intervention may reduce the gestural delay in children with ASD in their early childhood.

These studies highlight uses of robot-assisted interventions to teach social skills to children with ASD. The systematic review by Grossard and colleagues [24] reported excellent state of the art in the topic ICT and autism care in the period of 2017 to 2018. They analyzed serious games and social robots. The authors noted children with ASD have a specific need for predictability, visual support, and a sequential presentation of information, which aligns well with the use of social robots. They concluded that social robots offer clinicians new ways to interact and work with people with ASD.

### 3.2. Interventions with Serious Computer Games

Serious computer game was the other major category utilized in the studies evaluated. A game is a rule-based formal system with a variable and quantifiable outcome, where different outcomes are assigned different values, the player exerts effort in order to influence the outcome, the player feels attached to the outcome, and the consequences of the activity are optional and negotiable [47]. The term “serious games” denotes digital games serving serious purposes like education, training, advertising, research and health. Serious games, particularly adventure and shooter games, already play an important role in prevention and rehabilitation. Intelligent serious games are raising many hopes for developments in the educational field in the upcoming decades [48]. Computer games may be offered in combination with an internet connection or may stand alone. The combination of an internet connection, a multimedia environment featuring 2D/3D animation and virtual reality has led to the development of a plethora of serious simulation games intended for learning [49].

Seven of the 18 studies evaluated utilized a serious computer game intervention. Khowaja et al. [50] studied the use of serious games as an intervention to teach vocabulary words to children with ASD. After developing a game design framework specific to this population, an experimental evaluation prototype was introduced to examine its effectiveness in improving receptive identification of vocabulary terms. Pre- and post-evaluations demonstrated improvement in learning vocabulary terms among children with ASD after using the game, with retention of these terms at 2 weeks post-trial.

Aresti et al. [51] experimented with a computer game intervention to drive eye contact communication. This study of 20 children aged 3–8 years with ASD compared to neurotypically developing children utilized a 3-level computer game with preprogrammed automatic stops in the game requiring interaction and communication with the session leader to continue the game. An eye tracker measured eye contact and duration of eye contact. Reaction time was also evaluated. This intervention was delivered by an educationalist. The research was conducted by a multidisciplinary team of psychologists, educationalists, and engineers. This study took place in Spain. The authors concluded their study suggests usefulness of this serious game in cognitive rehabilitation of children with ASD.

Rice et al. [52] utilized a computer software program FaceSay™ and its face-processing instructions offered through three of its computer games to evaluate impact on emotion recognition, understanding another’s perspective, and social skill improvement with a sample of 31 elementary school HFA children in California, USA. Para-educators conducted the computer-aided intervention sessions. Results demonstrated significant differences between groups post-test in Affect Recognition score. The adjusted average (adj M) for the clinical group was 12.59, while the control group’s adjusted average was 8.50 [F (1.28) = 20.45, *p* < 0.001]. Mentalizing assessments for post-test Theory of Mind score after controlling for pretest score demonstrated these results: adj M = 12.39 clinical group vs. 16.85 control group [F (1.28) = 37.35, *p* < 0.001]. Teacher report assessing social impairment demonstrated significance post-test at *p* < 0.05 with adjusted mean 67.7 clinical group and 62.3 control group [F (1.28) = 4.523]. No significant differences were noted in teacher report for positive or negative interaction with peers. The study’s authors indicate their findings suggest computer technology can be effective in teaching children with ASD to understand the mental states of others, that FaceSay enhances ability to recognize emotion and understand another’s perspective, and that it shows opportunity to enhance these skills in the general school environment.

Boyd et al. [53] evaluated a collaborative iPad game’s effect on social relationships. Study participants included four dyads (eight children randomly assigned to pairs). The children were recruited from a school’s special day class for children with ASD in 3rd to 5th grades (ages 8–11 years). The study took place in California, USA. The purpose of this trial was to evaluate the relationship between specific game elements and level of intimacy in social relationships. The study intervention was “Zody” (Zody’s World: The Clock of Catastrophe) (SymPlay LLC, n.d.) [54], a collaborative iPad (Apple Inc., n.d.) [55] game with 4 mini-games designed to teach social skills. A cooperative mode was implemented during weeks 2 and 4 of the study, while LEGO^®^ play sets were utilized during weeks 1 and 3. The dependent variable was behaviors observed in the learning and practicing of social skills in domains of membership, partnership, and friendship. Results were obtained from observations collected by three different researchers along with participant interviews at the end of the study. Data went through several rounds of coding.

The domain Membership was supported by both the Zody iPad game and by a LEGO^®^ play set. The Partnership domain was similarly supported. Zody supported the Friendship domain through shared joy of winning, visual and auditory praise, earning points and “level up” when a game is won. Empathy was built into Zody as well as exhibited in opportunities to experience losses, offer commiseration, and hear auditory feedback commiseration scripts to later model. The authors concluded that collaborative games on tablets are effective as sustainable social skills intervention. Executive function skills, in particular working memory and cognitive flexibility were studied by de Vries et al. [56]. Executive function skills are cognitive processes that facilitate goal-directed behavior and flexibility. The impact of executive function training, notably working-memory and flexibility training offered through “Braingame Brian” [57] including game elements, were studied against a mock training control.

The study took place at the University of Amsterdam, Netherlands, and included 121 children with ASD, ages 8–12 years, with IQs greater than 80, and without seizure disorders. The participants were randomly assigned to the adaptive working memory training (N = 40), the adaptive cognitive flexibility training (N = 37), or the nonadaptive control mock training (N = 38). Following intervention, all children in all groups, including the mock control group, exhibited improvements in working memory, cognitive flexibility, attention, and parent ratings. No improvement was observed in inhibition. Statistically significant differences across groups were not achieved. The attrition rate was high: 26%. These results led the authors to conclude that the intervention in its present form was likely not justified for children with ASD.

A study conducted by Thomeer et al. [58] evaluated ASD symptoms, social skills, emotion encoding, and decoding skills in HFA children 7–12 years old, following the intervention of a computerized software program “Mind Reading: the Interactive Guide to Emotions” [59] and in vivo rehearsal. A sample of 43 was studied with 22 randomly assigned to the treatment group and 21 to the waitlist control group. The trial took place at a college campus in the USA and was conducted by the psychology discipline. The study was conducted to examine the primary author’s enhanced protocol for this cognitive based intervention. Emotion recognition (ER) in faces and in voices and emotion encoding and decoding skills were the primary measures assessed. ER in faces demonstrated significance at *p* < 0.001 in post-test and follow-up phases, indicating improvement in the clinical group over the control group. Effect sizes were large. ER in voices achieved similar results but limited to medium effect size at the follow-up phase with *p* = 0.006. Emotion encoding achieved significance as well. Differences between groups at post-test were t [40] = 2.33, *p* = 0.0125 (one-tail), d = 0.61 and at follow-up t [40] = 2.93, *p* = 0.003 (one-tail), d = 0.85, favoring the treatment group. Emotion decoding achieved significance between groups in the follow-up measure only, also favoring the treatment group. The authors asserted the study results suggest the “Mind Reading” protocol, inclusive of interactive software instructions, in vivo rehearsal, and behavioral reinforcement, was effective in improving all four primary measures assessed.

The final study evaluated was conducted by DeThorme et al. [60]. Utilizing a computerized feedback system developed from the software package VocSyl© against a traditional noncomputerized pacing board, speech was the target outcome in this study. Intervention arms of the study aimed to assist children to produce multisyllabic utterances. Participants included children with speech language impairments, age 2–8 years, diagnosed with ASD, Developmental Disability Unspecified, Cerebral Palsy, Childhood Apraxia, or Down Syndrome. Of the 18 child sample, six had ASD. The study took place at a Midwest university in the USA in Speech-Language clinic. Sessions were conducted by a graduate student clinician supervised by a certified speech-language pathologist. Outcomes evaluated included percent of target words at post intervention, at maintenance phase, and average number of new words achieved. Results demonstrated 58%, 60%, and 8–9 words, respectively, for the VocSyl group; 50%, 50%, and 7–8 words for the pacing board group; and 23%, 22%, and 3–4 words for the active control group.

## 4. Discussion

The reviewed 18 computer- and robot-assisted therapy studies address various aspects of social skills to help children with ASD overcome core deficits. They reveal noteworthy strengths, limitations, and implications. The strengths include study design, researcher attempts to address common issues known to people with autism in their study designs to reduce confounding variables and ease participant comfort, and inclusion of measures evaluating generalization of new skill in different environments and over time.

All studies included a research design with controlled comparison. Some method of randomization was achieved in most. These factors increase the validity of results by demonstrating the outcomes are specific to the intervention being studied if seen only in the experimental group and not in the control group. A variety of controlled study methods were utilized.

Another noted strength of the compiled studies was that many incorporated the subject’s real-world environment in their study protocol. This strategy is beneficial in a number of ways. It has the potential to reduce discomfort known to be common in children with ASD when faced with changes in their routine which often manifest as anxiety reactions producing negative behaviors [16,61]. Proactively minimizing variables such as these in the design of the research protocol not only keeps the subjects more comfortable but also has the potential to influence study validity by diminishing a confounding variable—anxiety—and its potential corruption of study results.

A third strength of the compiled studies is that many included generalization of the new skill as an outcome measure in addition to maintenance of that skill over time. Generalization is a common challenge in people with autism [1,12,16]. Generalization can be understood as taking what is learned in one setting or situation and being able to apply that knowledge to another setting or situation [12]. Most of the trials assessed generalization of the skills in alternative environments (e.g., at home or on the playground) or with people other than the researchers (siblings, peers, teachers, etc.). Maintenance of skill was assessed in several studies. Generalization and maintenance measurements suggest an intervention has sticking power and usefulness beyond the study environment itself.

The need to study a representative sample whose abilities and challenges are similar across the group is a factor that researchers must actively address when studying those diagnosed with autism, as this is a spectrum disorder. The compiled studies attempted to achieve this by utilizing inclusion/exclusion criteria for their samples, many using a standardized IQ score of 70 for U.S. studies and 80 for European & Middle East studies. In other trials that included participants from both IQ domains (some above 70, some below), the researchers reported results of the different subsets and established equivalence of experimental and control groups. These interventions assist in validity of results and generalizability to a specific subset of people with autism.

Limitations of the compiled studies were noted to include small sample sizes and the use of observation as a measurement method. Small sample sizes with fewer than 50 subjects were noted in all but 1 study. The deVries et al. [56] trial had a sample size of 121 participants. Aside from this study, small sample size may be influenced by the marked variance seen in individual abilities and limitations that make people with autism a challenging population to study. Researchers are pressed to find a common sample of subjects. Age bands often used to help define child samples are not necessarily representative of commonality in ASD samples, even when accounting for IQ score. This unfortunate situation may be due to the differing treatments, intensity, and duration of therapy offered to address ASD challenges in some children while others of the same age may have received no therapy at all. While the 18 studies had small or relatively small sample sizes, this may actually be representative of the researchers exercising strong sample selection methodology, serving as a study strength as well as a limitation.

A further limitation of several studies was reliance on researcher or parent observation of the outcome variable as a measurement method. This has the potential to produce less reliable results due to subjectivity and human error. While it was noted that the researchers incorporated efforts to minimize these risks by defining outcome terms and including inter-rater reliability measurement in the study protocols, nonetheless, the observation method of measuring results has the potential to contaminate findings. This literature review was also limited by its article selection methodology, exclusion criteria for publication, languages other than English, and the inability to access some documents to evaluate eligibility.

Implications of compiled results from the 18 studies evaluated generally reveal computer and robot assistance in social skills therapy interventions as early in its evolution. While several products being studied were still in the proof-of-concept stage or were iterations to enhance the technology or study it within a different sample population, their influence to drive improvement in social skill acquisition for people with autism is showing promise. Noted use of these products during therapies conducted by multiple disciplines working with these children further supports their usefulness. Awareness of the many computer- and robot-assisted therapies amongst educators, clinicians, funding sources, innovators, those with ASD, and their parents can promote continued application of these tools to help address core social skill challenges.

## 5. Conclusions

This paper intends to understand what IT platforms were being evaluated in computer- and robot-assisted social skills therapy for children with ASD, and further identify professional disciplines utilizing computer- and robot-assisted therapies, the outcomes, and intervention effectiveness. Robots and serious computer games were two noted types of IT tools utilized based on the reviewed literature. The number of robot studies published in 2018 compared to previous years suggests that the popularity of robots has increased recently.

It was noted that special education teachers, speech therapists, physical therapists, and psychologists were all evaluating the incorporation of these technologies into the therapy they provide. Outcomes being evaluated included emotion and face recognition, as well as communication methods including eye contact, speech vocalization, and pragmatic application. Social interactions, collaborative play behaviors, executive function abilities, task performance, imitation, social attention patterns and engagement, social relationship development, repetitive behaviors, and positive and negative affect were all studied.

The effectiveness of these interventions varied across the studies. The majority of studies demonstrated some level of encouraging results, despite several study’s limitations noted. A tool for further investigation is provided (Table A1). Organizing knowledge obtained from this review may assist professionals, parents, innovators, funders, and those with ASD to further examine each product. However, one research study concluded that its intervention in its present form was probably not suitable for children with ASD [56]; while another trial concluded there was limited effectiveness of its intervention [33]. Wide interest amongst stakeholders has the potential to promote enhanced capabilities of these technologies due to greater product use and further investment in the development of these computerized tools. Further studies are necessary to evaluate their effectiveness amongst adults with ASD and within unique subsets of the higher functioning autism population (e.g., those with above average or higher IQ scores). Advocating for the use of evidence-based therapies, including those that incorporate new technology, in an individual’s educational plan has the potential to produce benefits that last a lifetime.

## Appendix A

**Table A1:** Summary table of computer- and robot-assisted therapies for children with autism spectrum disorder (ASD).

Therapy Target	Author (Year)	Study Purpose	Additional Information
Serious Computer Games			
Eye contact, communication, social interaction	Aresti-Bartolome and Garcia-Zapirain (2015) [51]	Purpose: Assess how rehabilitation activities and supervised computer games added to a system designed for children with ASD can assist communication and interaction between children with ASD & professionals.	
Emotion recognition, mentalizing, social skills	Rice et al. (2015) [52]	Purpose: Determine the effects of FaceSay on ability of children with ASD to recognize emotions, understand another’s perspective, and improve their social skills compared with those not receiving the intervention.	Symbionica LLC. FaceSay™ Social Skills Software Games. http://www.facesay.com/
Executive function skills: Working memory, Cognitive flexibility, attention, & inhibition	de Vries et al. (2015) [56]	Purpose: Study 2 executive function training conditions: a working-memory (WM) training, and a cognitive flexibility training against a mock training control.	Prins, P. J., Ten Brink, E., Dovis, S., Ponsioen, A., Geurts, H. M., de Vries, M., & Van der Oord, S. (2013). “Braingame Brian”: Toward an executive function training program with game elements for children with ADHD and cognitive control problems. Games for Health Journal: Research, Development, and Clinical Applications, 2(1) doi:10.1089/g4h.2013.0004
Social relationship levels of intimacy	Boyd et al. (2015) [53]	Purpose: Explore the relationship between specific game elements and level of intimacy in social relationships for children with social skill challenges.	SymPlay LLC. Zody’s World: The Clock of Catastrophe. https://itunes.apple.com/us/app/zodys-world-clockcatastrophe/id821791253?mt=8
Communication Vocabulary learning	Khowaja et al. (2019) [50]	Purpose: Evaluate the use of a serious game developed using a game design framework developed for people with autism on improving vocabulary learning.	
Emotion recognition skills, ASD symptoms, social skills	Thomeer et al. (2015) [58]	Purpose: Evaluate the efficacy of a computer software (Mind Reading) and in vivo rehearsal treatment on the emotion decoding and encoding skills, autism symptoms, and social skills of children with HFASD.	Baron-Cohen, S., Golen, O., Wheelright, S., Hill, J. J. (2004). Mind Reading: The interactive guide to emotions. London: Jessica Kingsley Publishers.
Speech target productions	DeThorne et al. (2015) [60]	Purpose: Examine the effectiveness of 2 speech/language (S/L) interventions aimed at facilitating children’s multisyllabic productions: one incorporated in a novel computerized feedback system (VocSyl) & the other using a traditional noncomputerized pacing board.	Hailpern, J. M., Karahalios, K., DeThorne, L., Halle, J. (2010). Vocsyl: visualizing syllable production for children with ASD and speech delays. Assets ‘10. 297–298
Robots			
Collaborative play behavior	Huskens et al. (2015) [33]	Purpose: Investigate the effectiveness of a brief robot-mediated intervention based on Lego therapy on improving collaborative behaviors (i.e., interaction initiations, responses, & play together) between children with ASD & their siblings during play sessions.	Aldebaran Robotics. Nao. https://www.ald.softbankrobotics.com/en/cool-robots/nao LeGoff, D. B. (2004). Use of **LEGO**^®^ as a therapeutic medium for improving social competence. Journal of Autism and Developmental Disorders, 34, 557–571.
Sensory, negative and repetitive behaviors, and affective states	Srinivasan et al. (2015) [38]	Purpose: Systematically compare the effects of 8-wk long rhythm & robotic therapies to standard of care intervention on behavioral, affective, social communication & motor skills.	Aldebaran Robotics. Nao. (see reference above) WowWee. Rovio. http://wowwee.com/about/companyhistory
Social attention patterns	Srinivasan et al. (2016) [36]	Purpose: Study the effects of novel movement based interventions to current standard of care interventions for children with ASD; compare effects of human vs, robot trainer.	Aldebaran Robotics. Nao. (see reference above) WowWee. Rovio. (see reference above)
Social skills, Communication	Taheri et al. (2018a) [39]	Purpose: Evaluate robot-assisted clinical interventions with a therapist and social robots involving group games to study the impact on social & communication skills and stereotyped behaviors on fraternal twins with ASD. Pilot study.	Aldebaran Robotics. Nao. (see reference above) Robokind Company. Alice-R50. https://www.robokind.com/
Communication, Happiness	van Straten et al. (2018) [42]	Purpose: Playing puzzle game with a robot with required communication. The study examines the effects of intonation (normal vs. monotonous) combined with different bodily appearance of an embodied robot (mechanical vs. humanized) on treatment outcomes of robot-mediated therapy sessions for children with ASD.	This study was embedded in the BPicASSo project, which focuses on investigating the effectiveness of Pivotal Response Treatment for young children with ASD. Aldebaran Robotics. Nao. (see reference above)
Social interaction, acceptance of facial expression	Pour et al. (2018) [41]	Purpose: An initial attempt to develop a robotic platform for human-robot reciprocal interactions through facial expressions, investigating it acceptability and performance on children with ASD.	R50-Alice by Hanson RoboKind Company http://www.robokindrobots.com/
Social skills, social behaviors, communication, imitation, eye-hand coordination, classification, Joint attention and pointing	Taheri et al. (2018b) [40]	Purpose: Evaluate robot-assisted group games programs involving: Real-time imitation of the robot in upper body movements by the child Teaching imitation/motor skills by the robot to the children through individual/paired-group exercise and dances Playing a real xylophone Pointing to far/near points and showing the cards/objects by the robot/child Kinect-based recognition game and Classification of animals, fruits, places, and objects by pointing to different baskets on the screen Playing a developed Kinect-based virtual xylophone on the screen	Aldebaran Robotics. Nao. (see reference above) Robokind Company. Alice-R50 (see reference above)
Social skills, joint interaction, gaze orientation, pointing, vocal instruction	David et al. (2018) [43]	Purpose: Use of social robot to evaluate impact of. social cues on maintenance of joint attention by the child in a robot-child game	NAO developed by Softbank Robotics: Gouaillier D, Hugel V, Blazevic P, Kilner C, Monceaux J, Lafourcade P, Maisonnier B (2009) Mechatronic design of NAO humanoid. In: Robotics and automation, 2009. ICRA’09. IEEE international conference on 769–774. IEEE
Social skills, motor imitation, expressive/receptive language, spontaneous requests	Desideri et al. (2018) [44]	Purpose: Conduct a pilot test evaluating the impact of a social robot during education sessions to drive engagement and learning achievement	Aldebaran documentation. (2018). http://doc.aldebaran.com/2-1/family/robots/index_robots.html#all-robots.
Communication, sight word instruction, imitation, gazing, pointing, clapping	Saadatzi et al. (2018) [45]	Purpose: Evaluate a tutoring system featuring virtual teacher instructing sight words, and a humanoid robot emulating a peer upon the child’s word acquisition, retention, and maintenance performance	This study combined virtual reality technology (an avatar as virtual teacher) and social robotics for tutoring children with ASD.
Cognitive and motor skills, gestural learning	So et al. (2018) [46]	Purpose: Evaluate robot-based gestural training sessions utilizing social robot, NAO, whom children were told to imitate with goal to reduce the gestural delay in children with ASD in their early childhood.	NAO (see reference above)
